# An Overview on Recent Progress of Metal Oxide/Graphene/CNTs-Based Nanobiosensors

**DOI:** 10.1186/s11671-021-03519-w

**Published:** 2021-04-20

**Authors:** Ahmet Aykaç, Hazal Gergeroglu, Büşra Beşli, Emine Özge Akkaş, Ahmet Yavaş, Saadet Güler, Fethullah Güneş, Mustafa Erol

**Affiliations:** 1 Department of Engineering Sciences, Izmir Katip Çelebi University, 35620 Izmir, Turkey; 2 Department of Nanoscience and Nanotechnology, Izmir Katip Çelebi University, 35620 Izmir, Turkey; 3 Department of Nanoscience and Nanoengineering, Dokuz Eylul University, 35390 Izmir, Turkey; 4 Department of Material Science and Engineering, Izmir Katip Çelebi University, 35620 Izmir, Turkey; 5 Department of Metallurgical and Materials Engineering, Dokuz Eylul University, 35390 Izmir, Turkey

**Keywords:** Nanobiosensors, Metal oxides, Graphene, Carbon nanotubes, Nanohybrids biosensor scaffolds

## Abstract

Nanobiosensors are convenient, practical, and sensitive analyzers that detect chemical and biological agents and convert the results into meaningful data between a biologically active molecule and a recognition element immobilized on the surface of the signal transducer by a physicochemical detector. Due to their fast, accurate and reliable operating characteristics, nanobiosensors are widely used in clinical and nonclinical applications, bedside testing, medical textile industry, environmental monitoring, food safety, etc. They play an important role in such critical applications. Therefore, the design of the biosensing interface is essential in determining the performance of the nanobiosensor. The unique chemical and physical properties of nanomaterials have paved the way for new and improved sensing devices in biosensors. The growing demand for devices with improved sensing and selectivity capability, short response time, lower limit of detection, and low cost causes novel investigations on nanobiomaterials to be used as biosensor scaffolds. Among all other nanomaterials, studies on developing nanobiosensors based on metal oxide nanostructures, graphene and its derivatives, carbon nanotubes, and the widespread use of these nanomaterials as a hybrid structure have recently attracted attention. Nanohybrid structures created by combining these nanostructures will directly meet the future biosensors’ needs with their high electrocatalytic activities. This review addressed the recent developments on these nanomaterials and their derivatives, and their use as biosensor scaffolds. We reviewed these popular nanomaterials by evaluating them with comparative studies, tables, and charts.

## Introduction

A biosensor is a diagnostic device that converts signals from a biological analyte into a measurable and distinguishable electrical signal for a qualitative and/or a quantitative detection of the analyte that may embroiled with other physicochemical substances [1]. The first known biosensor was developed by Clark et al. [2] for the detection of oxygen, and the first amperometric enzyme electrode developed by Clark and Lyons [3] was an enzyme-based glucose biosensor. Over the years, enzyme-based, tissue-based, deoxyribonucleic acid (DNA)-based, and thermal, optical, electrochemical biosensor types have been developed. Biosensors give more stable and precise results than the traditional methods in some applications like clinical diagnosis, biomedical sector, food production, and analysis [2, 4]. Moreover, with such characteristics as specificity, selectivity, and cost savings with a simple operation, real-time analysis, and continuous use, various types of biosensors were developed rapidly through the second half of the century and have become widely used in related medical, environmental, and forensic fields [5]. Their intensive use in these critical application areas has emerged some anticipated features from a biosensor as high sensitivity, stability, high selectivity, long service life, repeatability, simplicity and cheapness, wide measuring range, and the fast response time [6].

**Fig. 1:**
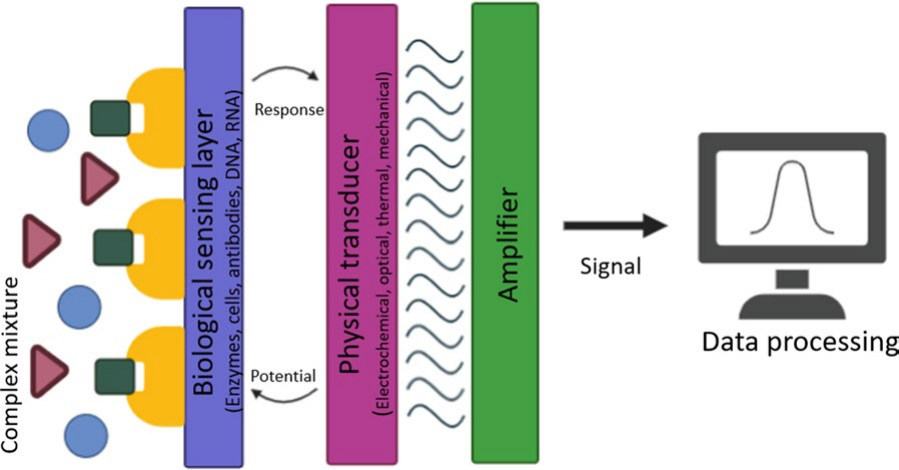
Schematic representation of biosensors

According to the International Union of Pure and Applied Chemistry (IUPAC), biosensors contain three main components: biological recognition element, transducer component, and electronic system that is often combined with a transducer. As integrated receptor–transducer devices, biosensors are able to provide selective quantitative or semiquantitative analytical information using a biological recognition element [7] (Fig. 1). Within this frame, nucleic acids, enzymes, antibodies, receptors, microorganisms, cells, tissues, and even biomimetic structures may be utilized as bioreceptor for biological detection.

The design of a biosensor is of great importance for a quick and convenient testing under any circumstance or any position that analyte may emerged. Within that design, transducer component materials have also a significant effect on the detection quality. The physical transducers vary significantly with the quantifiable signal source and utilize mostly optical and electrochemical systems [5]. The physicochemical, electronic/optical/electrochemical features of the material used as a physical transducer directly affect biosensors' performance. Additionally, biosensors' efficiency and effectiveness are determined by the matrices, mediators, and stabilizers used for enzyme immobilization. Therefore, the properties of the material from which the physical transducer component is produced play a critical role in obtaining such features as high signal stability and repeatability of biosensors and in their selectivity. Among aforementioned three components of a biosensor, this review mainly focuses on recent development on surface functionalization of transducer components using nanomaterials.

Transducers can be classified mainly into four classes: electrochemical, bioluminescent, piezoelectric, calorimetric, and optical. The surface of transducer can be modified by using many different functional materials so as to improve the sensor performance. Controlling the structure, morphology, and properties of these materials can also help in the same manner. Among these materials, nanosized materials, referred as nanomaterials, have a great potential to be crucial for the development of novel, adaptive, and highly sensitive biosensors for a broader application area with their unique size-dependent properties such as large surface area, improved electrical conductivity, and high chemical reactivity. Considering these extra-ordinary properties, nanomaterials have been one of the preferred candidates to meet the desired requirements for the construction of highly sensitive biosensors [6].

To be considered as a nanomaterial, at least in one dimension the size of a nanomaterial should be in between 1 and 100 nm [8]. Due to their highly minute size, in nanomaterials most of the atoms exist close to the surface or present on the surface. These nanoparticles (NPs), duly gaining remarkable features as enhanced physicochemical properties, higher surface area, shortened distance of electrons, bring out a significant difference compared to that of their bulk-sized counterparts. Thus, boosted performances would be maintained in the optical, thermal, electrical, and magnetic properties of those nanoscale materials to be highly effective for use as a biosensor component. Moreover, nanosized materials having higher surface area provide a suitable space for the immobilization of a sufficient number of bioreceptors on the surface of electrodes. Therefore, researchers have recently shown a great interest in the production, characterization, and use of nanomaterials for biosensor applications [9, 10].

Among all nanomaterials, MONs, graphene and its derivatives, and CNTs have stood out for their unique features [11, 12]. MONs exhibit significant catalytic properties due to their impressive morphological diversity, nontoxicity, and biocompatibility. It should also be noted that MONs provide a suitable structure for the immobilization of biomolecules.

Their crystal lattice allowing modification of the cell parameters and electrochemical properties due to quantum confinement effect, and the controllability of the bandgap by altering their surface properties affecting conductivity and chemical reactivity made them highly potential to be used as biosensing elements and differentiate MONs from their bulk counterparts [12, 13]. Moreover, to improve these properties further by forming a composite structure, MONs have recently been extensively combined with carbon nanomaterials such as graphene and CNTs to form a nanohybrid structure. Doing so improves the electrochemical reactivity for detection and diagnostic to meet the future requirements such as sensitivity and selectivity of a biosensor [14].

The hybridization of these carbon nanomaterials with MONs provides the production of advanced biosensors with one or more functions equipped with superior optical, magnetic, and electrical properties [14–16]. Graphene and its derivatives can be easily integrated with other nanomaterials to create nanohybrid materials to obtain desired electrochemical activity [13, 17, 18]. For instance, in many applications, graphene is regarded as a useful tool to promote electron transfer to proteins' redox response [19]. However, graphene's physical stability in the biological environment and its toxicity assessment to cells is still controversial [20–22]. On the other hand, CNTs, unlike graphene, have variant optical features due to their changing chirality making them advantageous compared to graphene in optical biosensing applications [23]. CNTs, having outstanding electrochemical ability, are readily chemically modifiable, and have high surface area to volume ratio like graphene [24]. In terms of surface properties, when exposed to an ambient, although graphene is exposed with its all volume due to its monolayer two-dimensional nature, this exposure is limited in the case of one-dimensional (1D) CNTs [25]. Additionally, it has been reported many times in previous studies that graphene has higher selectivity against interferences due to its excellent biomolecular sensing and signal-to-noise ratio properties compared to that of CNTs. It is mainly due to the metal-free graphitic edges of graphene with a high surface area. Nevertheless, problems as signal perturbation are exist in CNTs-based biosensors due to the presence of residual metal catalysts [25]. With all aforementioned aspects, nanohybrids formed by the combination of graphene and/or CNTs structures might play a vital role in the design of advanced biosensors, and compensation of disadvantages of both materials by forming a composite structure from them would overcome these problems and the detection could be maximized. Taking advantage of the cooperation created by the composite structure of MONs, graphene, and CNTs, it seems indispensable to provide an improved signal amplification and to prepare advanced bioaffinity strategies, resulting in development of improved biosensing devices to meet future requirements. Hence, within the scope of this review, it has been focused on recently realized MONs, graphene and CNTs-based biosensors. Moreover, the critical role of using these nanomaterials, not alone, but also together, in the production of biosensors with superior properties obtained by their combination has been discussed. By evaluating future expectations and challenges, we would like to put forward an alternative perspective for further studies.

## Metal Oxides Nanostructures-Based Biosensors

**Fig. 2:**
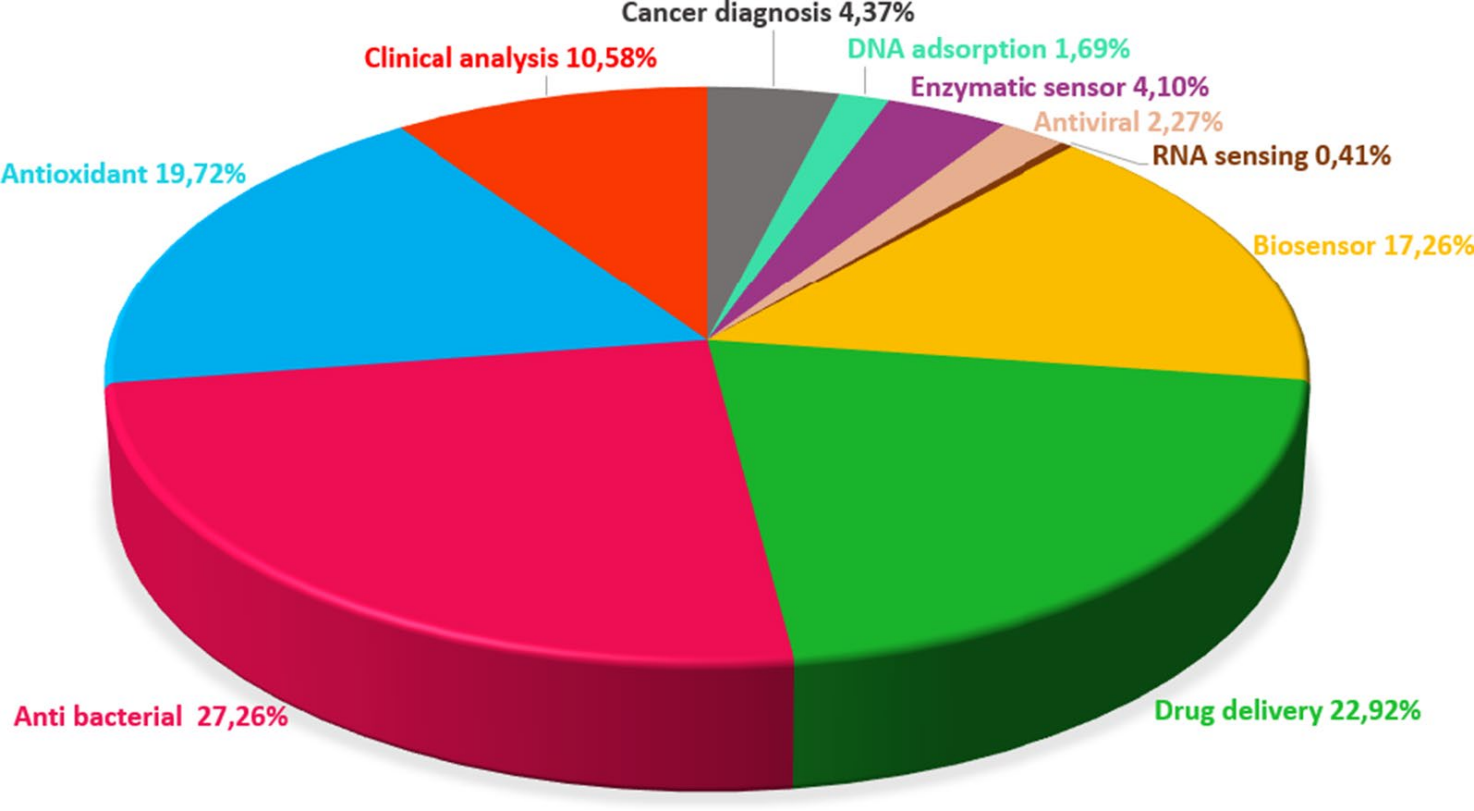
Pie chart showing the distribution of MONs in biomedical applications

Metal oxides (MOs) have been an essential candidates for sensor applications since initial biosensor studies in 1954 [26, 27]. MOs can be synthesized in various nanomorphologies such as NPs [28, 29], nanofibers [30], nanospheres (NSs) [31], nanorods [32], nanotubes and nanowires (NWs) [33], nanosheets [34, 35]. Besides morphological versatility, MONs offer some advantages: high surface/volume ratio, nontoxicity, good biocompatibility, chemical stability, excellent selectivity, electron and phonon limitation, high catalytic efficiency, and strong adsorption ability, physicochemical interface features [36–40]. Additionally, MONs can be produced via relatively easy and cost-effective methods such as radio frequency (RF) magnetron sputtering [41–43], thermal evaporation [44, 45], plasma-enhanced chemical vapor deposition (PECVD) [46, 47], molecular beam epitaxy [48], and solgel technique [49], electrochemical deposition process [50], and hydrothermal method [51]. These significant features have made MONs one of the most desired materials for biomedical applications and biosensor market. Publications on MONs from 2010 to 2020 were analyzed and are presented in Fig. 2 with a pie chart presented as the distribution of biomedical applications of MONs.

On the other hand, predominantly in recent years, various MONs such as ZnO, Fe_3_O_4_, CuO, NiO, TiO_2_, MgO have been continuously produced as versatile and functional biosensors for a long time [44, 52]. Among the MONs, ZnO and Fe_3_O_4_, due to their widespread applications, are considered to be prominent members in biosensor construction [53, 54].

### ZnO Nanostructures

ZnO nanostructures play an extensive role in the fabrication of novel nanostructured biosensors due to their unique properties including high isoelectric point (IEP ~ 9,5) [55], wide band-gap, useful electron communication feature, high chemical stability, good biocompatibility, and piezoelectricity. Especially, its high isoelectric point clearly explains why ZnO is the most prevalent metal oxide employed for biosensing technologies. Additionally, ZnO can be utilized in all clinical or nonclinical applications since it is environmentally friendly and safe material [53, 54, 56]. For instance, Akhtar et al. [57] developed a reagent-less optical biosensor based on the mechanism of fluorescence enhancement for the amyloid detection in the diagnosis of neurodegenerative diseases like Alzheimer's disease and insulin-dependent type II diabetes by utilizing flower-like ZnO nanostructures which have a greater surface area. Besides, ZnO nanoflower has been reported to be a good performance-enhancing material that provides a faster and cost-effective amyloid biosensor [57]. Further, a glucose biosensor using ZnO nanorod-based field-effect transistor (FET) related to wearable continuous glucose monitoring application for individuals with diabetes was fabricated by Zong and Zhu [54] via hydrothermal method. They achieved high-performance biosensor with a high sensitivity of 1.6 mA/µM cm^2^ with a tiny sensing area of 180 µm^2^ and a detection limit of 1 µM under the favor of the large surface-to-volume ratio of ZnO nanorods [54]. Sahyar et al. [58] developed a new Ag-doped ZnO NPs-based biosensor for early detection of meat spoilage. As a result of their analysis with an enzyme xanthine oxidase (XO)-modified electrode (nanoAg-ZnO/polypyrrole (PPy)/pencil graphite electrode), they stated that the enzyme biosensor they obtained showed high selectivity with 0.03μA/mM sensitivity and 0.07 μM low detection limit [58].

In another study, Yue et al. [59], successfully developed an ideal dopamine (DA) biosensor based on Au NPs-ZnO nanocone-arrays/graphene foam electrodes. In their characterizations, they proved that the electrode they modified has a high sensitivity (4.36 μA μM^−1^) and a low detection limit (0.04 μM, S/N = 3) in detecting DA. Furthermore, they reported that the ZnO nanocone-based electrode exhibited excellent selectivity, good reproducibility, and stability under uric acid (UA) interference. They also emphasized that the electrode has tremendous potential in medicine and health care [59]. In the same year, Qian et al. developed an electrochemical glucose detector using ZnO NPs. The sensor consists of a CeO_2_ nanowhisker decorated with ZnO NPs, and they stated that the ZnO/CeO_2_ nanocomposite structure has an extensive surface area, nontoxicity, and high electrocatalytic activity. The nanocomposite showed an extraordinary performance for detecting glucose with a linear range of 0.5–300 μM and a limit of detection (LOD) of 0.224 μM (40 ppb). They also emphasized that the nanocomposite sensor showed an excellent linear relationship between current signal intensity and glucose concentration (*R*^2^ = 0.99944) [60]. Another glucose biosensor was developed by Rafiee et al. [61] by combining graphene nanoplatelets (GNPs), known for their high conductivity and chemical stability, and ZnO NWs, known to be sensitive to glucose. In their study, they modified the structure of the device like a glucose biosensor by synthesizing ZnO NWs on thin films of GNPs in three different concentrations (0.5, 1, and 2 mg), defined as GNP1, GNP2, and GNP3. The system showed that the dual effect of ZnO NWs and GNPs led to the perfect improvement for an efficient glucose biosensor. For instance, they noted that for low glucose concentrations, the device's response increased as the amount of graphene in solution increased, and the sensor response time decreased with an increase in the number of GNPs. Moreover, they reported that long-term stability, namely consistent resistance to concentration relation, an important criterion for an ideal biosensor, was observed in samples modified with GNPs after exposure to 30 mg/dL glucose over 30 days. Consequently, they presented an ideal glucose biosensor with useful features: response time of 5 s, a detection range of 0.003–30,000 mg/dL, and long-term electrical stability [61]. In addition to these studies, some other recent studies using different ZnO nanostructures for the detection of various enzymes are given in Table 1.

**Table 1:** Selected recent biosensor studies based on nanomaterials including ZnO nanostructures

Nanomaterials and morphology	Types of biosensors	LOD	Sensitivity	Linear detection range	Analyte detected	Applications	References
ZnO nanoflower	Optical fluorescence	2.76 µg	1.388 mg/ml	-	Amyloids	Neurodegenerative disorders (Alzheimer, diabetes)	Akhtar et al. [57]
CuO-modified ZnO nanorods (NRs)	Electrochemical Amperometric	0.40 μM	2961.7μA mM^−1^ cm^−2^	0.001–8.45 mM	Glucose	Diabetes	Ahmad et al. [62]
ZnO NRs	Electrochemical-Potentiometric	NR	164.4 mV/decades	1 µM–10 mM	Glucose	Diabetes	Wahab et al. [63]
ZnO NRs	FET	1 µM	1.6 mA/(µM-cm^2^)	-	Glucose	Diabetes	Zong et al. [54]
Cu-doped ZnO NPs	Electrochemical Impedimetric	10^−9^ M	0.06 µF	10^−9^ M-10^−5^ M	Glucose	Diabetes	Mahmoud et al. [64]
ZnO/CuO/Co_3_O_4_ NPs	Electrochemical	9.7 ± 0.5 pM	36.98 μA μM^−1^ cm^−2^	0.05 nM–0.05 mM	Melamine	Food safety	Alam et al. [65]
ZnO Nanotube	Optical-Fluorescence	70 μM	3.5%·mM^−1^	0.1–15 mM	Glucose	Diabetes	Mai et al. [66]
ZnO nanosheets	FET	210 nM	0.27 mA/M/cm^2^	10 nM–1 mM	Formaldehyde	Life protection	Kim et al. [67]
CNT-embedded ZnO nanofiber	Electrochemical	5.368 zM	21.61 (KΩ μg^−1^ mL^−1^) cm^−2^	10 zM^–1^ µM	Atrazine	Environmental protection	Supraja et al. [68]
ZnO NWs/Graphene nanoplates	Electrochemical	0.003 mg/dL	–	0.003–30,000 mg/dL	Glucose	Diabetes	Rafiee et al. [61]
Flower-like ZnO nanosheets/Graphene	Electrochemical	0.0093 μM,	–	0.02–216 μM	Epinephrine	Clinical applications	Zhu et al. [69]
ZnO NRs/Carbon fibers	Electrochemical	0.45 fg/mL	6.09 μA/(g/mL)	1 fg/mL–1 μg/mL	Cortisol	Medical textile industry	Madhu et al. [70]
RuO_2_ doped ZnO NPs	Electrochemical Amperometric	96.0 ± 5.0 pM	5.42 μA μM^−1^ cm^−2^	0.1 nM–0.01 mM	l-glutamic acid	Food safety	Alam et al. [71]
ZnO quantum dots/BiOI nanoflower	Photoelectrochemical	3.3 pM	–	0.01- 500 nM	Histone acetyltransferase	Clinical applications	Chen et al. [72]

Considering the current studies shown in Table 1, it can be expressed that ZnO structures produced through numerous methods with varying morphologies, and it continues to be widely used due to its ease in integration into composite structures. Production alternatives and morphological versatility, as well as forming nanocomposite and nanohybrid structures with other nanomaterials, especially with carbon nanostructures, offer an extraordinary potential to ZnO structures in terms of meeting the expected properties with full efficiency in an ideal biosensor.

### Fe_3_O_4_ Nanostructures

In recent years, Fe_3_O_4_ nanostructure has aroused much interest in many promising applications, including biosensors, drug delivery, cell separation, and pharmacy, thanks to its superior properties such as good biocompatibility, low toxicity, superparamagnetism, catalytic activity, and the ease of preparation and modification process. Magnetic Fe_3_O_4_ NPs are appropriate for the immobilization of desired biomolecules such as enzymes [73–76] due to simple separation ability from the medium by its magnetic nature [77]. Fe_3_O_4_ magnetic NPs and their derivatives have been extensively used in biosensor technology, and various attractive studies have been discussed in the literature [75, 78]. In this context, Sanaeifar et al. [75] designed a new electrochemical biosensor for glucose detection. They evaluated the electrochemical performance of the nanocomposite prepared by dispersing Fe_3_O_4_ magnetic NPs, which were produced via the co-precipitation method in polyvinyl alcohol (PVA). They reported that Fe_3_O_4_ NPs in the PVA matrix, having excellent catalytic properties against immobilized glucose oxidase, increased the electron transfer rates between the enzyme and the electrode surface. The bioelectrode that prepared could measure glucose in the range of 5 $\times \hspace{0.17em}$10^−3^ to 30 mM with a sensitivity of 9.36 µA mM^−1^ and displayed a detection limit of less than 8 µM [75]. Dong et al. [79] developed Ag/Fe_3_O_4_ core–shell NSs-based sensors, produced via simple solvothermal approach, to be used in the detection of hydrazine for environmental protection. They reported that the high-performance hydrazine sensor has a 2 s response time, a linear range of 0.25–3400 µm, a sensitivity of 270 μA mM^− 1^ cm^− 2^, and a detection limit of 0.06 μM. Comparing the figures, a hydrazine sensor that is far superior to other sensors in the literature developed [79].

In another study, Sriram et al. [80] developed Fe_3_O_4_ NSs/reduced graphene oxide (rGO) nanocomposite to detect UA in urine and blood serum samples. As a result of their electrochemical analysis, Fe_3_O_4_ NSs/reduced graphene oxide (rGO) nanocomposites, with high stability and repeatability, showed an excellent electrochemical reduction peak. Moreover, they emphasized that the linear range of the UA sensor they developed was between 0.02 and 783.6 µM, and the LOD was 0.12 nM [80]. Likewise, a new biosensor for DA detection by combining graphene oxide (GO) and Fe_3_O_4_ was developed by Cai et al. [81]. In their study, they successfully synthesized Fe_3_O_4_/GO/pristine graphene (PG) ternary composite by dispersion and co-precipitation methods. Later, they deposited the nanocomposite on to the working electrode, glassy carbon electrode (GCE), by dropping technique. The highest peak current is recorded for Fe_3_O_4_/GO/PG structures in cyclic voltammograms (CVs). Similarly, they reported that the highest peak current in DA presence belongs to Fe_3_O_4_/GO/PG/GCE sample. They also highlighted an increase in the peak current for the Fe_3_O_4_/GO/PG/GCE sample due to increased DA concentration. Finally Cai et al. stated that the electrochemical sensor could effectively be used in DA detection [81]. Some representative studies on Fe_3_O_4_ nanostructures as a biosensor component are given in Table 2.

**Table 2:** Selected recent biosensor studies based on nanomaterials with Fe_3_O_4_ nanostructures

Nanomaterials and Morphology	Types of biosensor	LOD	Sensitivity	Linear detection range	Analyte detected	Applications	References
Fe_3_O_4_ NPs	Electrochemical-Amperometric	8 µM	9.36µA mM^−1^	5 × 10^−3^–30 mM	Glucose	NR	Sanaeifar et al. [75]
Fe_3_O_4/_Graphene/Pt flowers nanocomposite	Electrochemical Amperometric	1.58 µM	6.875 µA/mM	0.1 ~ 2.4 mM	H_2_O_2_	Clinical and nonclinical applications	Zhao et al. [82]
Fe_3_O_4/_rGO nanocomposite	Electrochemical	106.5 μA mM^−1^	2.645 μA mM^−1^	0.5–10 mM	Glucose	Diabetes	Wang et al. [83]
Fe_3_O_4 /_CNTs/PPy/Pd NPs	Electrochemical	1.417 × 10^−9^ M	–	2.247 × 10^−9^ M–2.752 × 10^−7^ M	Triclosan	Clinical and nonclinical applications	Zheng et al. [84]
Fe_3_O_4_/Graphene/Chitosan	Electrochemical Voltammetric	0.08 µM	–	0.4–2.0 μM	Ammonium	Environmental protection	Yu et al. [85]
Hollow magnetic Pt/Fe_3_O_4_/C NSs	Electrochemical Amperometric	0.43 µM	48.8 nA µM^−1^ cm^−2^	0.5–60 µM	Sarcosine	Prostate cancer	Yang et al. [86]
Graphene quantum dots (GQDs)/Fe_3_O_4/_MoS_2_ nanosheets	Optical Fluorescence	1.19 nM	–	2–64 nM	Epithelial cell adhesion molecule	Cancer diagnosis	Cui et al. [87]
Fe_3_O_4_/Au core–shell NPs	Optical Colourimetric	2 μM	–	5.0–70.0 μM	Catechol	Environmental protection	Karami et al. [88]
Cyclodextrin (CD)/Multi-walled carbon nanotubes (MWCNTs) Fe_3_O_4_/Chitosan/MWCNTs	Electrochemical Amperometric	19.30 µM	23.59 µA mM^−1^ cm^−2^	40 µM– 1.04 mM	Glucose	Diabetes	Peng et al*. *[89]
PtTi/GO/Fe_3_O_4_/MWCNTs-Fe_3_O_4_ nanocomposite	Electrochemical Aptasensor	25.3 pg mL^−1^	–	0.05–100 ng mL^−1^	Penicillin	Clinical applications	Guo et al. [90]
Methylcellulose/GO/Fe_3_O_4_ nanocomposite hydrogel	Electrochemical Potentiometric	0.17 μM	0.9093 μA/μM	0.5–140 μM	UA	Clinical applications	Sohouli et al. [91]
Fe_3_O_4_/Au nanoflowers	Surface-enhanced Raman scattering (SERS) Aptasensor	0.40 pg·mL^−1^	–	0.0001–100 ng·mL^−1^	Aflatoxin B1	Food safety and quality	He et al. [92]
Fe_3_O_4_ Nanoroses/Mesoporous GO sheets	Electrochemical	0.1 mM	1183.6 μA·mM^−1^ ·cm^−2^	0.1–16 mM	Glucose	Food and biomedical industry	Yao et al. [93]
PPy-coated Fe_3_O_4_/MWCNTs	Electrochemical	0.0230 μM	-	21.3–201 μM	Atorvastatin	Clinical applications	Tavousi et al. [94]

Despite their superior properties, magnetic Fe_3_O_4_ nanostructures have restrictive problems in biosensor and biological applications. Due to their high surface energy, chemical reactivity, and strong magnetic interactions, they are incredibly prone to agglomeration, creating difficulties in stabilizing Fe_3_O_4_ magnetic nanostructures. To overcome this problem, the surface of Fe_3_O_4_ nanostructures is coated with the polymer layers [95]. However, coating the surface with the polymer may decrease efficiency in terms of electrochemical biosensor applications. Thus, in stabilizing magnetic Fe_3_O_4_ nanostructures, biomolecules such as genes, cells, enzymes, proteins, and other essential nanostructures (graphene, CNTs, quantum dots, NPs, etc.) can be used. Therefore, it can be predicted that complex nanohybrid and nanocomposite systems based on magnetic Fe_3_O_4_ nanostructures will become a phenomenon in producing new generation biosensors in the future.

After all, MOs-based biosensors incorporating various nanostructures present unique and novel functions in practical and industrial applications. Nanostructures of MOs strongly impact devising highly sensitive, rapid, and stable biosensors due to their peerless properties. Besides, each kind of nanostructures and oxides of metals include its advantages. Hence, new advancements in sensing devices are likely to take place in biotechnology. Additionally, it is seen that nanocarbon structures have been given much space in recent studies, and MOs are used together with them. Therefore, the second part of this work will focus on the two most commonly used nanocarbon (graphene and CNTs) in biosensors.

## Graphene and Its Derivatives-Based Biosensors

**Fig. 3:**
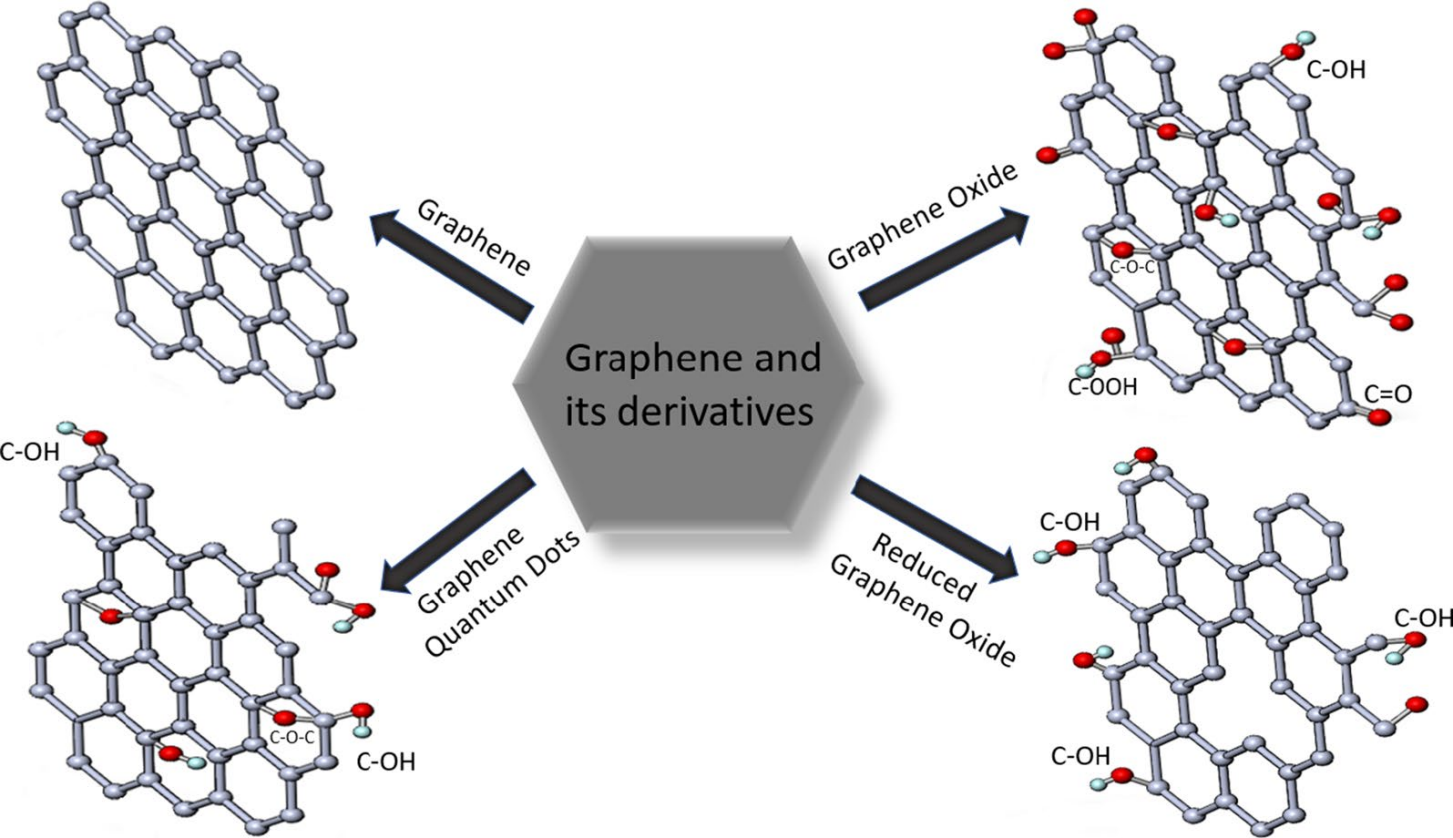
Structure of most popular graphene-based materials

Graphene is one of the most popular allotropes of carbon, just like graphite, CNTs, fullerene, diamond. It is a two-dimensional layer of sp^2^-hybridized carbon atoms. After the discovery of graphene by Geim and Novoselov, it has drawn huge attention worldwide in various disciplines such as transparent electrodes, energy storage, drug delivery, biosensors, supercapacitors, batteries, and catalysis [96, 97]. Graphene as many other nanomaterials can be synthesized by top-down (mechanical exfoliation, chemical exfoliation, and chemical synthesis) and bottom-up methods (pyrolysis, epitaxial growth, chemical vapor deposition (CVD)) [97]. Different production methods lead to the presence of numerous graphene-like materials such as graphene, GQDs, GO, rGO, graphene nanoribbons (GNRs), nanomesh, nanosheets [98]. The frequently used derivatives are shown in Fig. 3.

Graphene has good thermal conductivity (5000 W/mK), high electron mobility in room temperature (250,000 cm^2^/V s), large surface area (2630 m^2^/g), high modulus of elasticity (21 T Pa), and good electrical conductivity [99]. Furthermore, the atomic thickness of the graphene sheets and their high surface area provides material sensitivity against the changes in conditions. Thus, graphene's surface features, in which every atom can be directly contacted, make it sensitive to the environment. Therefore, it is an excellent candidate for sensor applications in comparison to the other materials [, , 4, 100, 101]. The last decade studies related to graphene and its derivatives were analyzed and are presented in Fig. 102 with a pie chart that presented the distribution of biomedical applications of graphene. It can be stated that researchers mostly focus on the field of biosensors due to the features of graphene mentioned above.

**Fig. 4:**
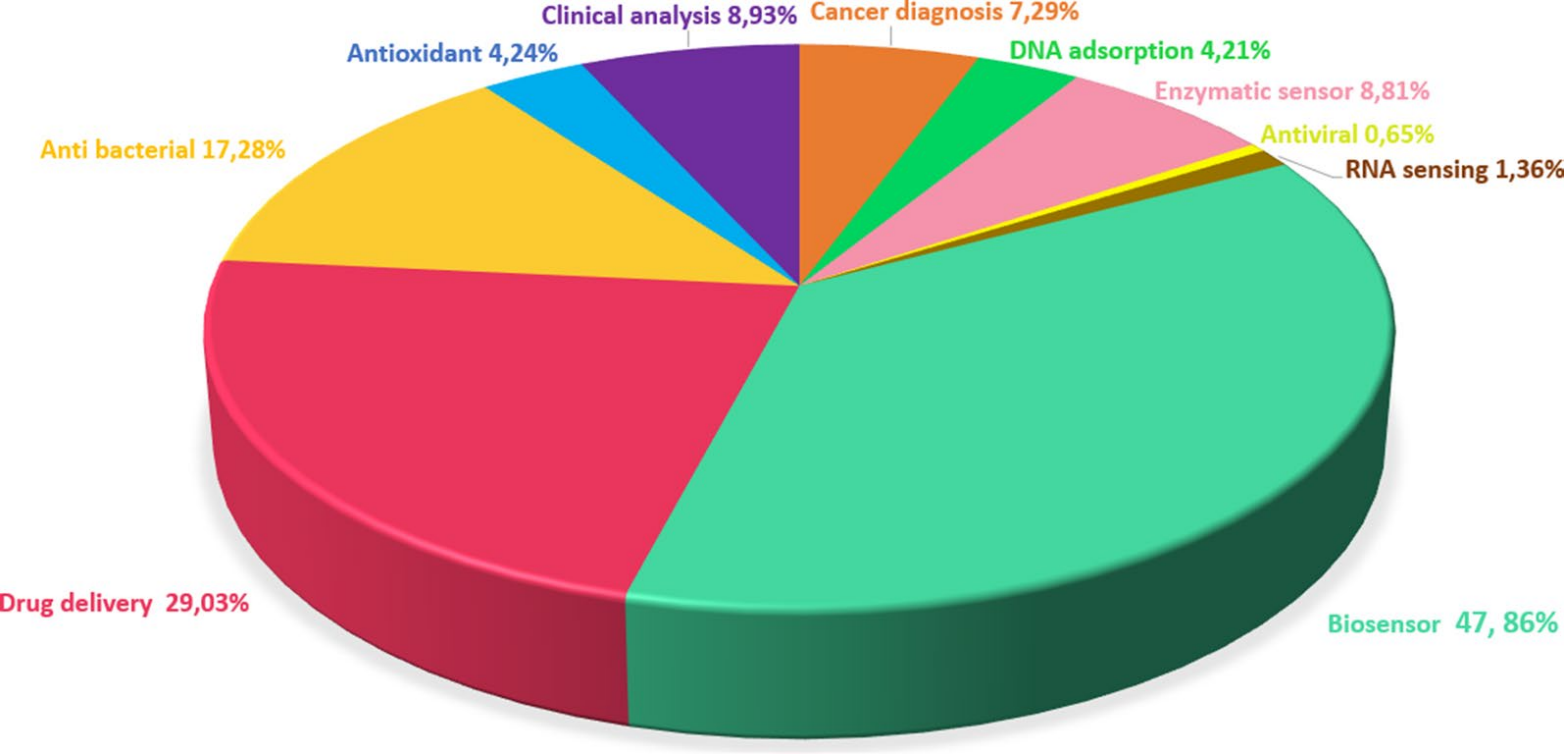
Pie chart showing the distribution of graphene in biomedical applications

**Fig. 5:**
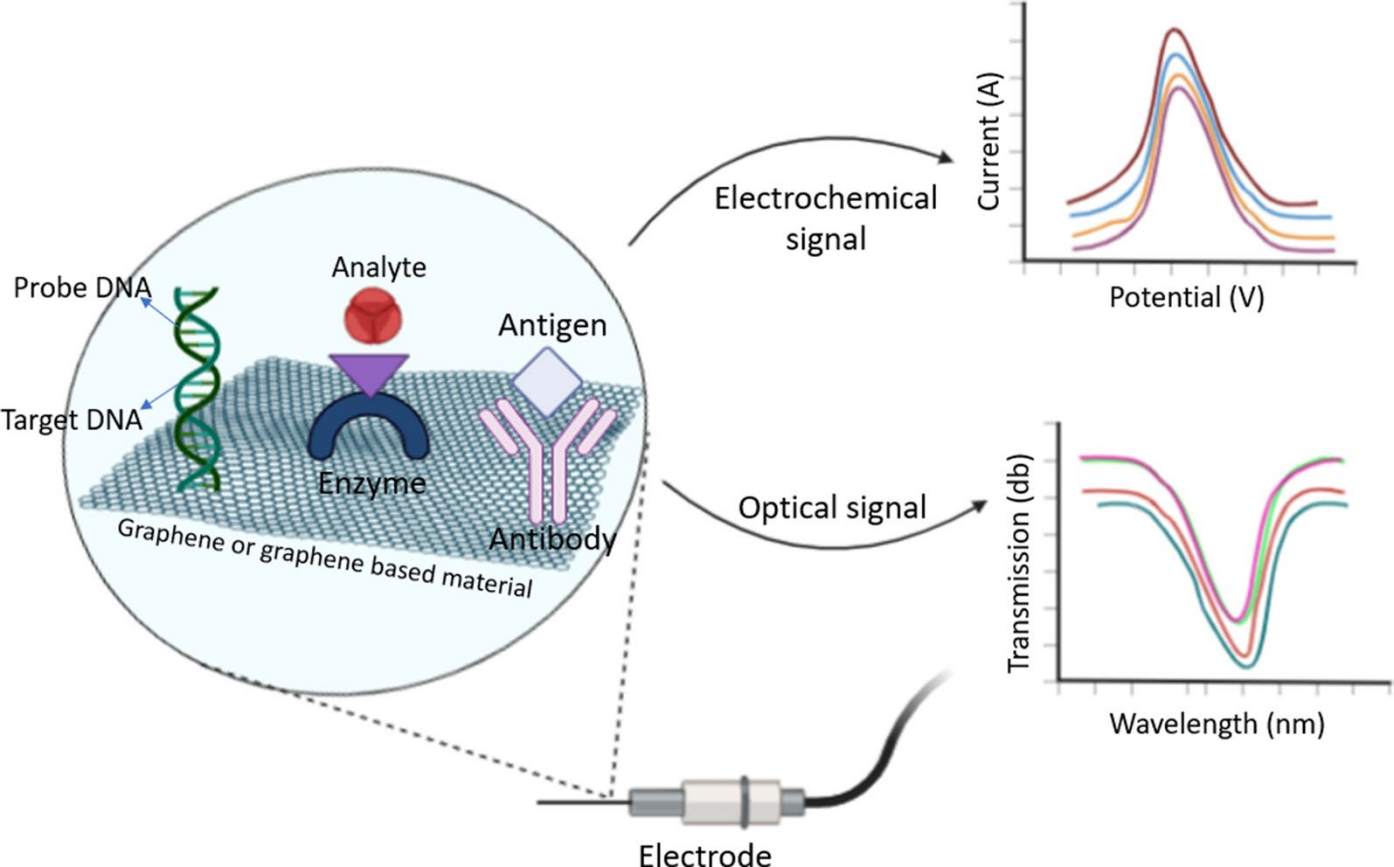
Representation of graphene-based biosensors and its mechanism

As mentioned in the first section, some biosensors are prepared by combining graphene and graphene derivatives with MONs. In this part of the review, we focus on biosensors based on graphene and its derivatives. A general representation and mechanism of graphene-based biosensors are shown in Fig. 5. Here, analytes interacting with the functional group (s) on the graphene surface, and electrochemical, optical, or other outputs can be obtained based on this interaction [96, 97, 103]. For instance, Mani et al. [104] developed a ternary nanobiocomposite based on rGO nanoribbons/MWCNTs/chitosan for sensitive and selective detection of H_2_O_2_ and NO_2_^−^. They explored the beneficial properties of the biosensor in contact lens cleaning solution and meat sample. They reported that for H_2_O_2_, the nanobiocomposite-based sensor had a sensitivity of 0.616 µAµM^−1^ cm^−2^, the detection limit of 1 nm, and a linear range of 0.001–1625 µM, while these values for NO_2_^−^, 0.643 µAµM^−1^ cm^−2^, 10 nm, and 0.01–1350 µM, respectively. Thus, they proved that the graphene-based sensor could be used effectively in medical applications and food safety [104]. Another graphene-based H_2_O_2_ sensor was prepared by Yin et al. [105]. In their study, Yin and colleagues synthesized conductive three-dimensional (3D) graphene aerogels (GA) decorated with Ni_3_N NPs using the hydrothermal method. As a result of their characterization, they showed that the Ni_3_N/GA composites they obtained could be applied not only for H_2_O_2_ but also for glucose determination. They reported that the Ni_3_N/GA-based electrode, in the determination of H_2_O_2_, demonstrated high electrochemical performance as the detection range of 5 µM–75.13 mM, the sensitivity of 101.9 µAmM^−1^ cm^−2^, and a low detection limit of 1.80 µM. Moreover, for glucose determination, they emphasized that the designed electrode has a detection range of 0.1–7645.3 µM, a detection limit of 0.04 µM, and a sensitivity of 905.6 µA mM^−1^ cm^−2^ [105].

It can be said that recently there has been intense interest in graphene-based biosensors for the practical detection of glucose Table 3. For instance, Đurđić et al. [106] successfully synthesized a single-use biosensor based on Bi_2_O_3_-decorated GNRs by co-precipitation. As a result of their characterization, they proved that the sensor they obtained had a detection limit of 0.07 mM, a linear range of 0.28–1.70 mM, and a sensitivity of 64.81 μA/mMcm^2^. Thus, they proposed that the graphene-based sensor could detect glucose in blood serum and urine samples reproducible and stable [106]. In the same year, a useful glucose biosensor was successfully designed by the single-pot hydrothermal synthesis of a 3D nitrogen-doped porous graphene hydrogel (NHGH) with NiCo_2_O_4_ nanoflowers (NHGH/NiCo_2_O_4_) by Lu and team. They modified the GCE with the nanocomposite they obtained and evaluated the modified electrode’s electrochemical performance in determining glucose. Firstly, they received CVs in 0.1 M NaOH solution, with a scan rate of 50 mV s^−1^, to examine the electrochemical catalytic performance. They reported that the NHGH/GCE has an increased oxidation peak current of 0.5 V than the weak anodic peak current of bare GCE. Moreover, in their study, they observed that the redox peak pair is visible, which indicates that the electrochemical activity of NHGH/NiCo_2_O_4_/GCE is highest compared to other electrodes. They attributed this improvement to graphene's extended surface area**,** good conductivity, and Co and Ni’s redox reactions. In addition, they showed the electrochemical catalytic performances of the electrodes in the 5.0 mM glucose addition. They interpreted NHGH/NiCo_2_O_4_/GCE with the highest peak current at 0.5 V as a clear indication that glucose oxidation could be better catalyzed than other electrodes due to the dual effect of NiCo_2_O_4_ and NHGH. They also reported that the peak currents increased linearly with increasing glucose concentration and the NHGH/NiCo_2_O_4_-based glucose sensor exhibited a broad linear relationship between peak current and glucose concentration in the range of 5 μM–2.6 mM and 2.6 mM–10.9 mM, respectively. Also, they emphasized that NHGH/NiCo_2_O_4_/GCE has a high sensitivity (2072 μA mM^− 1^ cm^− 2^) and a low detection limit (0.39 μM). As a result, they suggested using for a precise determination of glucose in real blood samples [107].

**Table 3:** Selected recent biosensor studies based on graphene and its derivatives

Nanomaterials and Morphology	Types of biosensors	LOD	Sensitivity	Linear detection range	Analyte detected	Applications	References
rGO/Ni/ZnO NRs arrays	Electrochemical Amperometric	0,15 µM	2030 µA mM^−1^ cm^−2^	0.5 µM–1.11 mM	Glucose	Clinical applications	Mazaheri et al. [108]
GO/MoS_2_ aerogel	Electrochemical	0.29 mM	3.36 µA/mM	2–20 mM	Glucose	Clinical and nonclinical applications	Jeong et al. [109]
Graphene flakes/Ni	Electrochemical	1 µM	2213 µA mM^−1^ cm^−2^	1–1150 µM	Glucose	Clinical diagnosis	Wu et al. [110]
rGO/Au and Pt alloy NPs	Electrochemical	5 µm	48 µA/mM cm^2^	50 mV/s–150 mV/s	Glucose	Medical Textile Industry	Xuan et al. [111]
GO	Optical Fiber	-	0,24 nm/mM = 1,33 nm/(mg/ml)	-	Glucose	Bioengineering applications	Jiang et al. [112]
rGO/Ni NPs/Gelatin methacryloyl (GelMA)	Electrochemical	0.005 µM = 5 nm	0.056 mAmM^−1^	0.15 µM-10 mM	Glucose	Clinical and nonclinical applications	Darvishi et al. [113]
Chemically rGO	Electrochemical Amperometric	5. 10^−8^ M	0,0040 AM^−1^	1.5 × 10^−7^–3.0 × 10^−6^ M	H_2_O_2_	Medical applications	Nieto et al. [114]
GO/Ag/Au NPs	Electrochemical	0,001 µg/ml	0.084 µA m/cm^2^ µg/ml	0.01 − 5000 μg/mL	Cholesterol	Clinical diagnosis	Huang et al. [115]
Graphene/Poly Diphenylamine (PDPA)/Phosphotungstic acid (PTA)	Electrochemical Amperometric	0.1 μM	1.085 μA/μM cm^2^	1–13 μM	Urease	Electrocatalytic applications	Muthusankar et al. [116]
Graphene/MoS_2_/TiO_2_/SiO_2_ layers	Surface plasmon resonance	-	82.83 Deg-RIU^−1^	-	Formalin	Food safety	Hossain et al. [117]
N-Doped Graphene/Polyaniline (PANI)/DNA-Functionalized CNTs	Electrochemical	14 nM	–	0.02-1 μM	DA	Molecular diagnosis	Keteklahijani et al. [118]
Monolayer graphene/Au NPs	Electrochemical	0.1 nM	–	0.0005–5000 μM	Glucose	Diabetes	Yuan et al. [119]
NiO-N-doped carbon/rGO microspheres	Electrochemical	70.9 nM	4254μAmM^−1^ cm^−2^	0.5 μM − 20.0 μM	Glucose	Food analysis and clinical diagnosis	Zhang et al. [34, 35]
Ionic liquid-functionalized graphene/CNTs	Electrochemical	3.99 × 10^−7^ mol/L	53.89 μA mmol/L^−1^ cm^−2^	0.004–5 mmol/L	Glucose	Diabetes	Zou et al. [120]
GO nanofibers/Cu nanoflower-decorated Au NPs	Electrochemical	0.018 μM	–	0.001–0.1 mM	Glucose	Clinical applications	Baek et al. [121]
GO/NiO Films/Au NPs	Electrochemical	7.64 µM	57.16 mV/decade	0.01 mM–100 mM	Urease	Clinical and nonclinical applications	Nien et al. [122]
Thermally rGO	Electrochemical Amperometric	0.02 mM	2.3 ± 0.1 µA cm^−2^ mM^−1^	0.2–12.0 mM	Urease	Medical Technologies	Razumiene et al. [123]
GNPs/graphitized nanodiamond	Electrochemical	0.005 mg/mL	806.3 μA(mg mL^−1^)^−1^ cm^−2)^	0.1–0.9 mg/mL	Urease	Food analysis	Kumar et al. [124]

As seen in Table 3, graphene and its derivatives have become an indispensable building block for biosensor applications, because of its excellent properties. Considering the studies performed recently Table 3, it is remarkable that graphene and its derivatives are used in hybrid nanostructures with MONs to improve biosensors' sensitivity and reproducibility. Additionally, MONs/graphene synergy should be evaluated to obtain multifunctional biosensors and achieve high electrocatalytic activity. Moreover, graphene can be easily combined with other nanocarbons such as CNTs. Therefore, rich edge density and highly beneficial edge defects for creating enzymatic biosensors can be obtained.

## Carbon Nanotubes-Based Biosensors

**Fig. 6:**
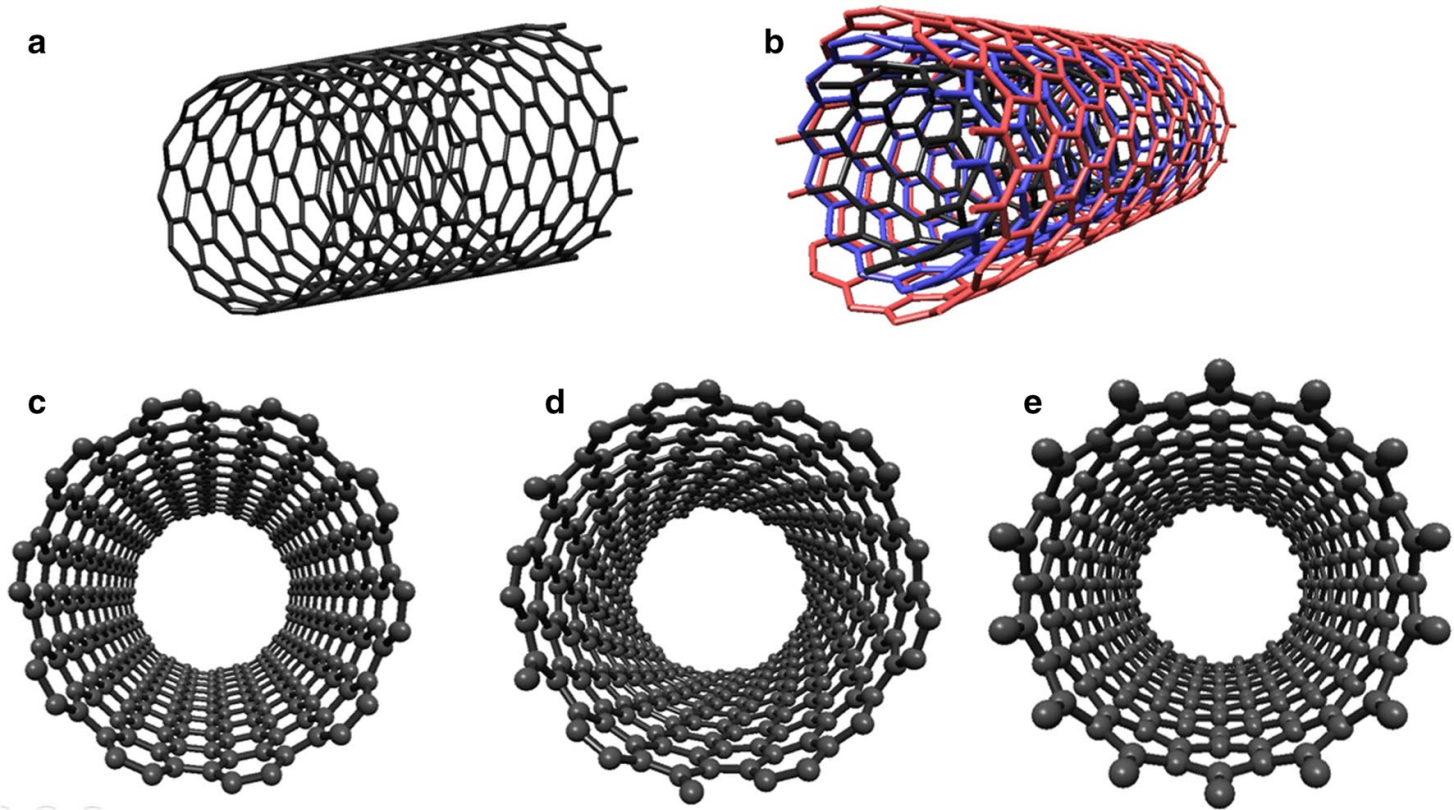
The classification of the CNTs of **a** SWCNT, **b** MWCNT; Schematic representation of three typical types of SWCNTs **c** Armchair (10, 10), **d** Chiral (13, 6), and **e** Zigzag (14, 0)

CNT's, discovered by Iijima in 1991, can be conceived as the formation of a graphene layer into a cylinder. CNTs can be categorized in general two types as single-walled carbon nanotubes (SWCNTs) Fig. 6a and MWCNTs Fig. 6b [125]. The diameter and wrapping angle determine the physical features of the CNTs by chirality and the (n, m) index [126–128]. According to the (n,m) index, CNTs can exhibit metal or semiconductor behavior [129–132],depending on chirality, SWCNTs may be classified in three different ways: (1) *m* = *n* is the armchair nanotube Fig. 6c, (2) *n* > *m* and if *m* = 0 is the chiral nanotube Fig. 6d, and *m* = 0 is the zig-zag nanotube Fig. 6e. CNTs display the semiconductive behavior in their nature, but for a given (*n*, *m*) SWNT, when (2*n* + *m*)/3 is an integer, the CNTs will be metallic. Thus, it can be claimed that all armchair nanotubes are metallic [130]. Therefore, the ability to control chirality during production means to control the electronic features of CNTs, which provides a great advantage in biosensor applications. Several different methods have been proposed to synthesize CNTs in recent years. However, there are three main synthesis techniques (arc discharge, laser ablation, and CVD for CNTs production [133]. Compared to arc-discharge and laser ablation methods, CVD is the most effective method for simple and cost-effective controlling the chirality of CNTs [133, 134].

**Fig. 7:**
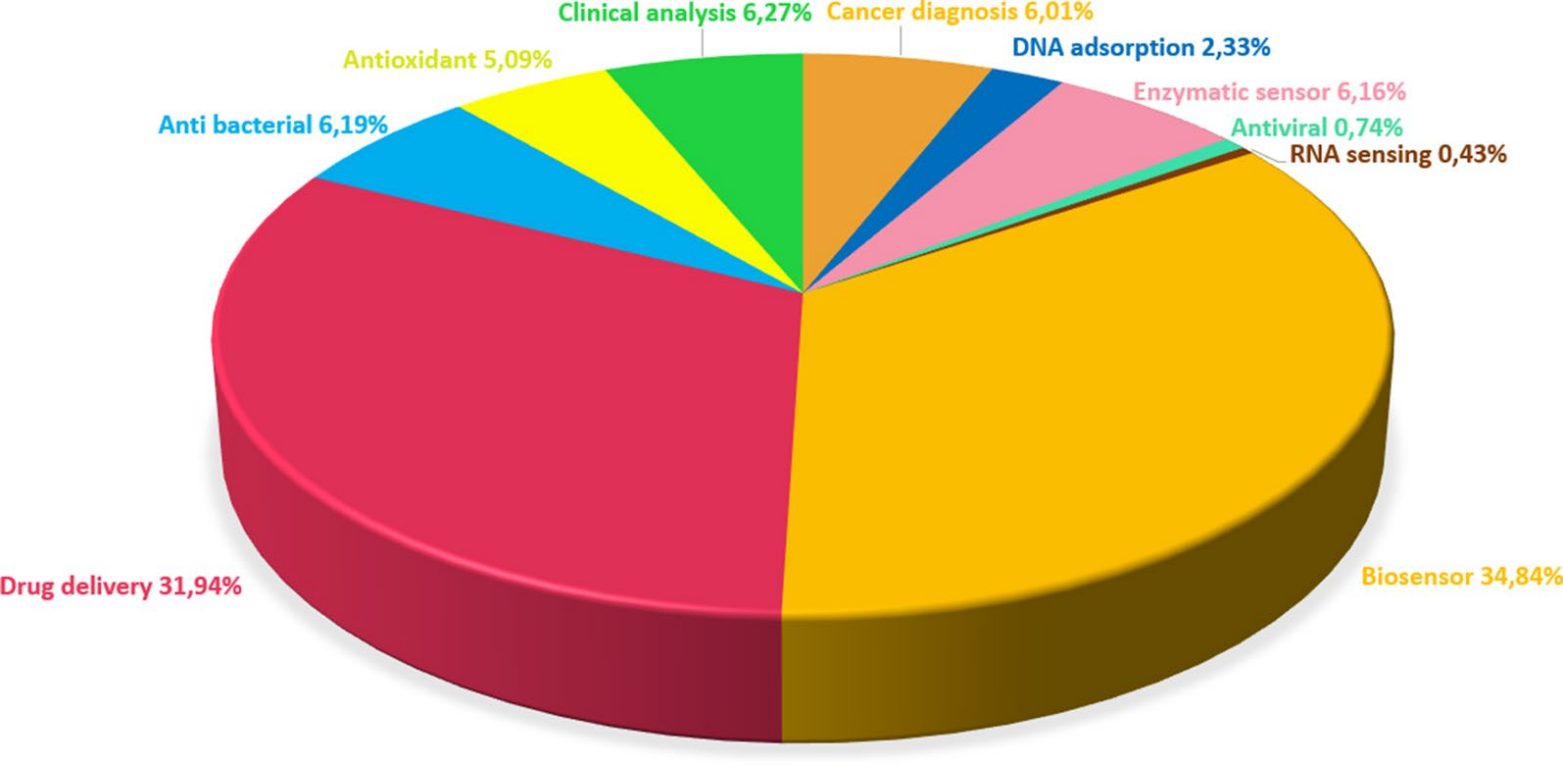
Pie chart showing the distribution of CNTs in biomedical applications

The ends and sidewalls of the CNTs can be easily modified by the addition of virtually any desired chemical species. CNTs can be excellent transducers in nanoscale sensors owing to their significant sensitivity. Additionally, CNTs have very favorable properties for transmitting electrical signals generated upon recognition of a target and therefore play an essential role in the final development of enzyme-based biosensors [135]. Moreover, CNTs with small size, fast response times, and excellent electrochemical properties are equal or superior to most other electrodes with their ions, metabolites, and protein biomarkers [136]. As a result of their unique tubular nanostructures with extensive length and diameter ratios, CNTs are desirable materials in applying electrochemical biosensors due to their excellent electrochemical stability, great mechanical flexibility, rapid electron transport, and unique thermal conductivity [137, 133]. CNTs are also widely used in tissue engineering and drug delivery systems to improve electrical and mechanical features after being functionalized to ensure their biocompatibility and conjugated with organic compounds or metallic NPs. [138]. Studies on CNTs from 2010 to 2020 were analyzed and are presented in Fig. 7 as a pie chart that shows the distribution of biomedical applications of CNTs.

CNTs, as with graphene and its derivatives, also make important contributions to the development of biosensors with higher sensitivity and selectivity by hybridizing with MONs. Researchers have recently focused on the production and characterization of new nanobiosensors that can combine the unique properties of CNTs with the superior properties of metal NPs. For instance, Rahman et al. [139] designed the Fe_3_O_4_-decorated CNTs based 3-methoxyphenyl (3-MP) biosensor for environmental protection applications. Fe_3_O_4_/CNTs nanocomposites synthesized by wet-chemical method and coated the nanocomposite on the GCE surface as a thin layer. Then, they evaluated the electrochemical performance of the modified electrodes by I-V characterization and reported that the Fe_3_O_4_/CNT-based electrode showed a wide detection range (90.0 pM–90.0 mM), low detection limit (1.0 pM), and high sensitivity (9 × 10^−4^ μA μM^−1^ cm^−2^) in detecting dangerous phenol [139]. Similarly, for environmental protection, MWCNT/TiO_2_/chitosan-based biosensor was developed by Fotouhi et al. [140] to detect dihydroxy benzene isomers released into the environment from the chemical and pharmaceutical industries. Fotouhi et al. reported that they performed the simultaneous determination of hydroquinone (HQ), catechol (CC), and resorcinol (RS), causing pollution in real water samples by the MWCNTs-based sensor. Additionally, they indicated the detection limits (*S*/*N* = 3) of HQ, CC and RS, as 0.06 μmol d m^−3^, 0.07 μmol d m^−3^, and 0.52 μmol d m^−3^, and the linear response ranges are between 0.4–276.0 μmol d m^−3^, 0.4–159.0 μmol d m^−3^, and 3.0–657 μmol d m^−3^, respectively [140].

Besides environmental protection, biosensor designs of CNTs for clinical applications have recently become extremely interesting Table 4. For instance, Zhu et al. [141] obtained the buckypaper containing two layers: purified SWCNTs and SWCNTs decorated with NiO, by helium arc discharge method. Later, as a result of their analysis to evaluate its electrochemical performance, they showed that glucose biosensor has a broad linear range (0.1–9 mM), high sensitivity (2701 μA mM^−1^ cm^−2^), and fast response time (< 2.5 s) [141]. Barthwal and Singh [142] designed a ZnO/MWCNTs nanocomposite biosensor to detect urea in their study. They indicated that the ZnO/MWCNTs-based sensor has the highest detection characteristics compared to the ZnO and MWCNTs-based sensor. Also, they emphasized that the nanocomposite's sensitivity containing 2% MWCNTs is less than 10 s, and the detection limit is 10 ppm [142]. In the same year, Guan et al. successfully developed a CNTs-based hybrid nanocomposite as an electrochemical biosensor for simultaneous high-sensitivity detection of DA and UA. In their study, they reported that the most extensive (Δ*E*_p_ = 144 mV) and highest oxidation current was observed in the electrode modified with CNTs-based nanohybrid. Additionally, they investigated the simultaneous detection of DA and UA in nanohybrid-modified GCE via differential pulse voltammetry (DPV). They showed that the anodic peak current response of the nanohybrid/GCE increased linearly due to the increase in DA concentration. Also, they obtained a similar observation for the UA concentration. They emphasized that the concentration range for both target analytes is 2–150 μM. As a result, they reported that the limit of DA and UA detection values was 0.37 μM and 0.61 μM, respectively [143].

**Table 4:** Selected recent biosensor studies based on CNTs

Nanomaterials and morphology	Types of biosensors	LOD	Sensitivity	Lineer detection range	Analyte detected	Applications	References
CNTs/Dendrimer-encapsulated Pt nanoclusters	Electrochemical Non-enzymatic	0.8 µM	987.5 µA mM^−1^ cm^−2^	0.003–0.4 mM	H_2_O_2_	Clinical applications	Liu and Ding [144]
CNTs/Pd-Co NPs	Electrochemical Non-enzymatic	0.3 µM	–	1 µM–1.11 mM	H_2_O_2_	Clinical and nonclinical analysis	Huang et al. [145]
		1 µM	75.4 µA mM^−1^ cm^−2^	10 µM–2.4 mM	Glucose	Diabetes	
CNTs/mucin composite	Electrochemical Amperometric	3 μM	0.44 ± 0.01 mA M^−1^	0.002–3.2 mM	Glucose	Diabetes	Comba et al. [146]
MWCNTs/Graphene/Poly(diallyl dimethyl ammonium chloride) (PDADMAC)	Electrochemical	4.40 μM	–	5–50 μM	UA/Hypoxanthine	Clinical applications	Si et al. [147]
CNTs/Co_3_O_4_/TiO_2_	Photoelectrochemical	0.16 μM	0.3 μAmM − 1 cm − 2	0–4 mM	Glucose	Diabetes	Çakıroğlu and Özacar [148]
CNTs/NiO/Poly(3,4-ethylenedioxythiophene) (PEDOT)	Electrochemical	0.026 µM	7.36 μA μM^−1^ cm^−2^	0.03–20 µM	DA	Clinical applications	Sun et al. [149]
		0.063 µM	3.04 μA μM^−1^ cm^−2^	0.3–35 µM	Serotonin		
		0.210 µM	0.92 μA μM^−1^ cm^−2^	1–41 µM	Tryptophan		
CNTs/ZnFe_2_O_4_	Colorimetric	0.58 μM	–	0.8–250 μM	Glucose	Diabetes	Wang et al. [150, 151]
MWCNTs/GQDs/Au NPs/Chitosan	Electrochemiluminescence	64 nM	–	0.1–5000 μM	Glucose	Diabetes	Wang et al. [150, 151]
MWCNTs/CdO NPs	Electrochemical	4.0 pM	25.7911 μA μM cm^−2^	0.01 nM–0.1 mM	M-tolyl hydrazine hydrochloride	Environmental safety	Rahman et al. [152]
MWCNTs/PANI	Electrochemical	10 µM	0.38 μA mM^−1^ cm^−2^	10–50 µM	Urease	Disease diagnosis	Bao et al. [153]
CNTs/ZnO NWs	Electrochemical Amperometric	3.3 ng/µl	–	3.3 ng/µl-3.3 mg/µl	Urine albumin	Medical applications	Tabatabaei et al. [154]
MWCNTs/Fe_3_O_4_/PANI	Electrochemical Amperometric	67 µM	–	1.0–25.0 mM	Urease	Food analysis	Singh et al. [155]
MWCNTs/AuNPs/PPy	Electrochemical Impedimetric	0.1 × 10^−3^ M	10.12 μA mM^−1^ cm^−2^	2 × 10^−3^–8 × 10^−3^ M	Cholesterol	Clinical applications	Alagappan et al. [156]
MWCNTs/Ni(OH)_2_	Electrochemical	0.095 μmol L^−1^	–	0.5–26 μmol L^−1^	Folic acid (vitamin B_9_)	Food safety	Winiarski et al. [157]
Carboxylated SWCNTs/Molecularly imprinted polymer (MIP)/Chitosan	Electrochemical	0.025 ng mL^−1^	–	0.04–7.6 ng mL^−1^	Semicarbazide	Food safety	Yu et al. [158]
MWCNTs/GQDs	Electrochemical	0.87 nM	–	0.005–100.0 μM	DA	Clinical applications	Huang et al. [159]
MWCNTs/Co-based Metal organic framework (MOFs)/Au NPs	Electrochemical	0.4 μM	0.223 μA μM^−1^	1–1000 μM	Nitrite	Environmental safety	Lei et al. [160]
SWCNTs	Electrochemical Field-effect transistor	0.01 mM	0.4–0.6 µA/mM	0.01–2 mM	Glucose	Clinical and nonclinical applications	Pandey et al. [161]
SWCNTs/Pt–Pd NiO NPs	Electrochemical	3.0 nM	0.2267 µA/µM	0.008–350 µM	Daunorubicin	Clinical applications	Alizadeh et al. [162]
		0.1 µM		0.5–330 µM	Tamoxifen		
CNTs/Pt NPs/Au: Ru NPs	Electrochemical Amperometric	0.068 mM	0.2347 nA/(μM mm^2^)	1 − 10 mM	Glucose	Prediabetes and diabetes	Nguyen et al. [163]
MWCNTs/CoS NPs	Electrochemical	5 μM	15 mA M ^−1^ cm^−2^	8 μM-1.5 mM	Glucose	Diabetes	Li et al. [164]
CNTs/peptide-decorated Au NPs	Electrochemical	6 pg/mL	–	0.01–1000 ng/nL	Matrix metalloproteinase-7	Cancer diagnosis	Palomar et al. [165]
CNTs/Fe_3_O_4_ NPs/rGO	Electrochemical Amperometric	0.54 μM	–	1 − 50 μM	Antipsychotic drug trifluoperazine	Medical applications	Ognjanovic et al. [166]

Studies on increasing the efficiency of CNTs-based biosensors in different application areas by hybridizing with MONs and graphene and graphene derivatives and improving their properties are of great interest Table 4. The higher electrochemical activity and higher conductivity of nanohybrid structures designed with CNTs-based electrochemical sensors can be considered a result of the inherent properties of CNTs. On the other hand, one of the features that limit the use of CNTs in biosensor applications is that they are not dissolved in most solvents. Also, it has low biocompatibility and, in some cases, toxicity. To overcome these problems, combining different functional groups on the surface and end caps of CNTs with MONs, and applying surface modifications can be considered as a solution.

Additionally, due to the integration of CNTs with graphene and its derivatives, it is possible to create more active sites for biomolecules due to strong binding interactions. Another advantage of CNTs/graphene hybrid structure is that it allows biosensors to respond in a shorter time due to their higher electron transfer rate. Thus, in the next generation of biosensors to be developed in the future, it seems inevitable to achieve high sensitivity and selectivity, simultaneous target biomolecule detection by benefiting from the dually effect of CNTs with MONs or other nanocarbons such as graphene and its derivatives.

## Conclusion and Outlook

Biosensors and bioelectrodes play a crucial role in environmental monitoring, food safety, the medical textile industry, drug discovery and analysis, clinical and nonclinical applications. With the recent COVID-19 pandemic, fast responsive, reusable, cheap and highly selective biosensors became crucial for the fight against infectious diseases to be taken under control. For the design of a biosensor, the material used in transducer component and to functionalize transducer surfaces has an explicit effect on the results with aforementioned properties obtained from a biosensor. Within this frame, for the improvement of the properties of these devices, nanomaterials have been extensively used and their expanded surface area, ability to adapt to the surface modifications for the use of any type of analyte, and such extraordinary nanosize-dependent properties brought them one-step ahead unprecedently in the production of an ideal biosensor.

With this motivation, this paper presents an overview on recent developments in hybrid nanosystems created by the combined use of MONs, graphene, and CNTs. Numerous efforts have been made to create biosensors with improved sensitivity and selectivity to detect biomolecules with the help of these nanostructures. Obviously, apart from each of these materials’ unique characteristics, the multiple effect of hybrid design of them is a key point in obtaining a higher performance biosensor. Combining these nanostructures to create a hybrid design improves the biosensor's electrocatalytic activity, its electron transfer rate, and enables more active sites to allow two or more biomolecules to be detected, simultaneously. It also meets other desired functions expected from an ideal biosensor, such as stability, long shelf life, repeatability, wide measuring range, fast response time for next-generation biosensor applications. However, there are compelling factors in combining these three trending nanomaterials, such as the control on agglomeration tendency, cytotoxicity, the choice of the right concentration, and the extensive optimization of conditions to improve purity and these materials better integration with each other. Therefore, there are still open allowance for improvements to be made for the preparation of nanomaterials and their composite structures. Furthermore, for an onsite diagnosis of an analyte, having a major impact for biosensors for medical applications, it is important to have a quick and reliable result in a cost-effective way. For this purpose, nanomaterials used in biosensors might be modified to facilitate diagnosis with more delicate sensing especially for the biomarkers of some diseases with a very minute concentration at their early stages. For gaining and improving such features, graphene, CNTs and MONs, should be produced with minimum catalyst impurities, high crystallinity, and in massive amounts in a cost-effective way. They should also be engineered for their density of states and the structure of bonds for tailoring a better electron transport properties. Within this review, a combination of nanostructures that help to develop an accurate 'future biosensor' mechanism was proposed and expectations as sensitivity, superior selectivity, low limit of detection, real-time sensing with multi-functional properties were summarized.

## Data Availability

Not applicable.

## Abbreviations

1D: One-dimensional
3D: Three-dimensional
CD: Cyclodextrin
CVD: Chemical vapor deposition
CVs: Cyclic voltammograms
DA: Dopamine
DNA: Deoxyribonucleic acid
DPV: Differential pulse voltammetry
FET: Field-effect transistor
GCE: Glassy carbon electrode
GelMA: Gelatin methacryloyl
GNPs: Graphene nanoplatelets
GNRs: Graphene nanoribbons
GO: Graphene oxide
GQDs: Graphene quantum dots
LOD: Limit of detection
MIP: Molecularly imprinted polymer
MOFs: Metal organic frameworks
MOs: Metal oxides
MWCNTs: Multi-walled carbon nanotubes
NHGH: Nitrogen-doped porous graphene hydrogel
NPs: Nanoparticles
NRs: Nanorods
NSs: Nanospheres
NWs: Nanowires
PANI: Polyaniline
PDADMAC: Poly(diallyl dimethyl ammonium chloride)
PDPA: Poly Diphenylamine
PECVD: Plasma-enhanced chemical vapor deposition
PEDOT: Poly(3,4-ethylenedioxythiophene)
PG: Pristine graphene
PPy: Polypyrrole
PTA: Phosphotungstic acid
PVA: Polyvinyl alcohol
RF: Radio frequency
rGO: Reduced graphene oxide
SERS: Surface-enhanced Raman scattering
SWCNTs: Single-walled carbon nanotubes
UA: Uric acid
XO: Xanthine oxidase
